# From microbiome profiling to precision medicine: diagnostic and therapeutic potential in gastrointestinal disorders: current evidence, challenges, and future directions

**DOI:** 10.1097/MS9.0000000000005027

**Published:** 2026-04-27

**Authors:** Laiba Arif, Mohammad Moosa Abbasi, Ahmed Asad Raza, Abedin Samadi

**Affiliations:** a Department of Medicine, Jinnah Sindh Medical University, Karachi, Pakistan; b Department of Medicine, Kabul University of Medical Sciences Abu Ali Sina, Kabul, Afghanistan

**Keywords:** 16S rRNA sequencing, colorectal cancer, dysbiosis, fecal microbiota transplantation, gastrointestinal disorders, gut microbiome, inflammatory bowel disease, irritable bowel syndrome, microbiome profiling, precision therapeutics

## Abstract

Gastrointestinal (GI) disorders, affecting millions globally (approximately 1.5 billion people with IBS alone), impose a significant healthcare burden and remain challenging to diagnose and manage. Current approaches are often invasive or symptom based, highlighting an urgent need for more precise and personalized strategies. The gut microbiome may offer novel diagnostic biomarkers and therapeutic targets, potentially transforming patient care. It supports GI and systemic health via metabolism, immune modulation, and neurochemical signaling. The dysbiosis of the gut microbiota contributes significantly to the pathogenesis of various GI disorders, including inflammatory bowel disease (IBD), irritable bowel syndrome (IBS), colorectal cancer (CRC), and small intestinal bacterial overgrowth. This narrative review critically evaluates the diagnostic potential of microbiome profiling and its clinical applications in developing personalized therapeutic strategies. We examine cutting-edge techniques such as 16S rRNA sequencing, metagenomics, and metabolomics, and discuss how dietary modulation, precision probiotics, and fecal microbiota transplantation are being increasingly used to reshape gut microbial composition. However, it is critical to note that while microbiome alterations show consistent associations with GI diseases, current evidence remains largely observational and associative. To date, no microbiome-based test has achieved regulatory approval or clinical validation as a standalone diagnostic tool for IBD, IBS, or CRC, and therapeutic applications remain investigational with modest clinical benefits in select conditions. Additionally, we highlight the translational challenges of integrating microbiome-based diagnostics into mainstream clinical practice and propose future research imperatives. This review provides a balanced perspective on the promise and challenges of integrating microbiome-based approaches into clinical gastroenterology, while proposing actionable research priorities to guide future investigations toward clinically validated, patient-centered diagnostic, and therapeutic solutions.

## Introduction

### The burden and complexity of gut disorders

**Figure 1.**
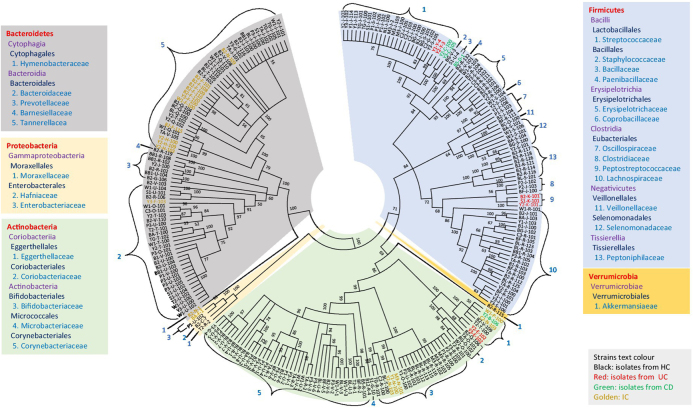
Diversity of novel gut isolates from disease-associated (CD, UC, and IC) and HC fecal samples. The cladogram represents the taxonomic classification of all 245 potential novel strains comprising at least 100 novel species from 27 families down to the family level. The cladogram is color-coded, as depicted in the boxes according to five phyla. The text color of the strains demonstrates the sources of isolates (CD, UC, IC, and HC)[10].

Gastrointestinal (GI) disorders, such as irritable bowel syndrome (IBS), inflammatory bowel disease (IBD) and colorectal cancer (CRC), are the major contributors of progressive bowel damage affecting millions worldwide and significantly impact patient quality of life. Recent global analysis estimates that IBS affects approximately 15% of the world’s population, with the highest prevalence observed in South America (18.9%) and the lowest in South Asia (11.0%)[1]. The global incidence of IBD nearly doubled between 1990 and 2021, increasing from about 199 000 new cases to over 375 000 annually, affecting approximately 3.8 million people worldwide[2]. These numbers are expected to rise further by 2050 as the disease burden shifts toward developing nations. While CRC remains the third most commonly diagnosed cancer globally, its incidence has increased sharply in low- and middle-income regions due to westernized diets and sedentary lifestyles[3]. These disorders often present with heterogeneous symptoms like abdominal pain, altered bowel habits, bloating, unexplained weight loss, inflammation, and bleeding that often make diagnosis challenging as they overlap between these disorders. IBD represents a chronic inflammatory condition of the GI tract caused by an inappropriate immune response to intestinal microbiota, whereas IBS is a functional bowel disorder characterized primarily by dysregulation of gut–brain communication rather than mucosal inflammation. CRC, in contrast, arises from neoplastic transformation influenced by microbial dysbiosis, inflammation, and life style factors^[^4–6^]^. Furthermore, their multifactorial etiologies encompassing hereditary inclination, environmental influences, immunological reactions, and living patterns hinder diagnosis and treatment. The lack of mechanistic insight into the disease results in prolonged diagnosis and reliance on nonspecific therapies. Traditional approaches for diagnosing GI disorders like colonoscopy, symptom scoring, and clinical warning signs frequently fall short when it comes to uncovering the root causes of disease. While colonoscopy remains the go-to tool for directly viewing the colon’s lining. Even under optimal conditions, colonoscopy may miss approximately 6–12% of adenomas, particularly flat or subtle lesions, underscoring the limitations of morphology-based detection alone^[^7–9^]^. Correspondingly, symptom assessment tools used for diseases such as IBS and IBD often do not correlate with objective measures of inflammation or mucosal damage. This can lead to delayed diagnosis and inconsistent treatment outcomes. These challenges highlight critical gaps in current diagnostic practice and underscore the growing need for more precise, non-invasive tools. In this context, gut microbiome profiling has emerged as a complementary approach, offering insights into disease-specific microbial signatures and host–microbe interactions (Fig. 1)^[^10^]^.

HIGHLIGHTS

- Gut microbiome profiling enhances early diagnosis of gut disorders accurately.
- Personalized probiotics significantly improve chronic gastrointestinal symptoms.
- Diet modulation reshapes gut microbiota, reducing inflammation effectively.
- Fecal microbiota transplantation shows promise in chronic gut disease relief.
- Translational challenges in clinical microbiome integration need addressing.

This review provides a balanced assessment of current microbiome profiling methods and their potential to complement existing diagnostic modalities while informing the design of precision therapeutic approaches in GI medicine.

Table 1 highlights key best practices for microbiome research, emphasizing the need for standardized terminology, reliable sample handling, and consistent data management. Adhering to these guidelines improves accuracy, reproducibility, and comparability across studies.

**Table 1:** Microbiome-based diagnostic insights across gut and systemic disorders.

Gut disorders	Microbiome test methods	Key microbial signatures
Inflammatory bowel disease	16S rRNA, shotgun metagenomics, shotgun sequencing of stool samples	↓Firmicutes, ↑Proteobacteria,
		↑Faecalibacterium prausnitzii,
		↓Escherichia coli
Irritable bowel syndrome	16S rRNA, metabolomics, shotgun sequencing of stool samples	Altered Bacteroides, Firmicutes; ↓F. prausnitzii
Colorectal cancer	Stool-based metagenomics and metabolomics; mucosal biopsy microbiome analysis	↑Fusobacterium nucleatum, Peptostreptococcus, ↓Firmicutes
Small intestinal bacterial overgrowth	Breath test (hydrogen methane), 16S rRNA sequencing of duodenal aspirates or stool samples	Overgrowth of E. coli, Klebsiella and anaerobes
Autism spectrum disorder	Network on gut microbiome shortgun data	Altered Prevotella and Bacteroides ratios
Major depressive disorder	Metagenomics	Enrichment of Bifidobacterium and Lactobacillus species
Type 2 diabetes	Logistic regression with multi-omics data	↑Akkermansia muciniphila and Roseburia levels

### The clinical challenge: GI disorders and unmet diagnostic needs

The global incidence of IBD is increasing, particularly in newly industrialized nations, contributing to increasing disability-adjusted life years and healthcare expenditures[11]. IBS, by contrast, is a functional bowel disorder characterized by abdominal pain and altered bowel habits without overt inflammation or structural abnormalities, yet it significantly impairs quality of life and productivity. CRC remains one of the leading causes of cancer-related mortality worldwide, with early detection being critical to improving survival outcomes.

Despite advances in diagnostic technologies, current methods often fall short in identifying the underlying pathophysiology of these disorders. Symptom-based assessment tools for IBS and IBD frequently lack correlation with objective measures of disease activity such as mucosal inflammation or histological damage, leading to delayed diagnosis and suboptimal treatment outcomes. Alarm symptoms such as rectal bleeding or unintentional weight loss demonstrate limited sensitivity and specificity when used in isolation, hindering the differentiation between functional and organic disorders.

These limitations underscore a critical gap in clinical practice: the need for non-invasive, objective, and mechanistically informed diagnostic tools that can identify disease at earlier stages, predict disease progression, and guide personalized therapeutic strategies. In this context, gut microbiome profiling has emerged as a promising complementary approach, offering insights into disease-specific microbial signatures, host–microbe interactions, and metabolic dysregulation that are often overlooked by traditional diagnostic modalities.

### Overview of the human gut microbiome

Microorganisms have been around for millions of years and may be found in almost every habitat. The human microbiome is a varied collection of bacteria that live in harmony with the body, particularly in the large intestine. There, these microorganisms consume dietary carbohydrates and convert them into a variety of helpful fermentation products[12]. Human microbiomes are critical to health throughout the lifespan and are increasingly recognized and investigated for their involvement in metabolic, immunological, and neurological processes[13]. Moreover interactions between the host and the microbiome have an influence on a number of physiological processes as well as complex disease states[14]. They also assist to combat infection and inflammation by interacting with and modifying our immune systems[15]. The gut bacterial community regulates several aspects of metabolic diseases. This control is dependent, among other things, on the microbiota’s synthesis of a vast range of metabolites and their interactions with receptors on host cells, which may activate or inhibit signaling pathways and be either beneficial or harmful to the host’s health[16]. Also the gut microbiota and the brain are closely related, and there is ample evidence that the gut microbiota is crucial to the brain’s physiological functions. The microbiota–gut–brain axis, which includes immunological, endocrine, neurological, and metabolic signaling pathways, is the mechanism via which the gut microbiota and the host brain communicate reciprocally as a result of long-term symbiosis and co-evolution. Recent studies have linked the gut microbiota to several diseases, including multiple sclerosis, Parkinson’s disease, Alzheimer’s disease, and autism spectrum disorder[17].

### Rationale and objectives of this review

This narrative review critically evaluates the current state of knowledge regarding the diagnostic and therapeutic potential of gut microbiome profiling in GI disorders. Specifically, we seek to:

1. Examine the technological landscape of microbiome profiling, including 16S ribosomal RNA (rRNA) gene sequencing, metagenomics, and metabolomics, and critically assess their capabilities and limitations in identifying disease-associated microbial signatures.
2. Critically appraise the evidence supporting microbiome-based biomarkers for the diagnosis and monitoring of IBD, IBS, and CRC, including sensitivity, specificity, clinical validation status, and applicability compared to existing diagnostic standards such as colonoscopy, fecal immunochemical testing (FIT), and serological markers.
3. Evaluate microbiome-guided therapeutic interventions, including dietary modulation, precision probiotics, prebiotics, synbiotics, and fecal microbiota transplantation (FMT), emphasizing evidence from randomized controlled trials (RCTs), systematic reviews, and meta-analyses, including efficacy, safety concerns, regulatory challenges, and standardization issues.
4. Identify and analyze key translational challenges, including inter-individual microbiome variability, lack of standardized methodologies for sample collection and bioinformatics analysis, the distinction between causality and association, limited longitudinal data, and the need for multi-omics integration.
5. Discuss regulatory and safety considerations specific to microbiome-based products, including certification, verification, and validation requirements for clinical implementation.
6. Propose actionable future research priorities and articulate a vision for integrating microbiome science into precision medicine frameworks for GI care, including strategies to overcome current limitations.

By synthesizing current evidence and critically analyzing conflicting findings, methodological biases, gaps, and inconsistencies in the literature, this review aspires to serve as a comprehensive, evidence-based resource for clinicians, researchers, and policymakers navigating the rapidly evolving field of microbiome-based diagnostics and therapeutics.

### Methodology

This narrative review was conducted to comprehensively evaluate and synthesize current literature on the diagnostic and therapeutic implications of the human gut microbiome in GI disorders such as IBD, IBS, and CRC. A structured electronic search was performed using PubMed, Scopus, and Google Scholar for studies published between 2010 and May 2025, employing key terms such as “gut microbiome,” “gastrointestinal disorders,” “16S rRNA sequencing,” “metagenomics,” “fecal microbiota transplantation,” “microbiome biomarkers,” and “precision probiotics.” Additional articles were identified through manual screening of bibliographies from key reviews and clinical guidelines to ensure comprehensive coverage of relevant evidence. Studies were selected if they were published in peer-reviewed journals, written in English, and focused on human subjects or clinically relevant human microbiome models. Eligible literature included original research articles, systematic reviews, meta-analyses, and clinical guidelines addressing diagnostic uses of microbiome profiling or microbiome-guided therapeutic interventions. Excluded were non-English publications, animal-only studies without translational value, conference abstracts, editorials, and articles lacking empirical data or clear clinical implications. Studies focused exclusively on microbiomes of non-GI sites such as oral, skin, or vaginal microbiota were also omitted. Screening of titles and abstracts was performed independently by two reviewers, followed by full-text evaluation, and any disagreement was resolved by discussion. Study quality was assessed based on design, sample size, diagnostic clarity, microbiome methods, and confounder control. Particular weight was given to studies using standardized protocols, validated assays, and reproducible analytical methods. Evidence strength was categorized narratively as high (systematic reviews, meta-analyses, large RCTs), moderate (observational or cohort studies with sound methodology), or low (pilot or small-scale studies). Data were synthesized narratively and organized thematically into diagnostic and therapeutic domains, emphasizing clinically significant microbial signatures, sequencing technologies, and translational potential. This approach allowed critical integration of heterogeneous findings and identification of methodological gaps, biases, and future research needs. The review process adhered to the 2025 TITAN Guidelines on transparency and responsible use of artificial intelligence in research and publication practices[18].

## Technological landscape: Tools to decode the microbiome

The rapid evolution of sequencing and multi-omics technologies has revolutionized our understanding of the gut microbiome’s composition, function, and clinical relevance. These approaches now enable high-resolution profiling of microbial communities, allowing researchers and clinicians to move beyond descriptive ecology toward functional and diagnostic insight[19].

### 16S rRNA gene sequencing

16S rRNA gene sequencing is a widely used method for profiling bacterial community composition by sequencing conserved and hypervariable regions of the 16S rRNA gene. As our understanding grows, so does the potential to diagnose, monitor, and even predict health outcomes – using nothing more than genetic traces left behind by microbes. In order to study the taxonomic composition of a bacterial community, the sequencing of the 16S rRNA gene has been the gold standard[20]. The development of DNA sequencing technologies identifies what types of bacteria are present in a sample on the 16S rRNA gene. This gene is about 1500 base pairs long and contains nine variable regions that help tell different bacteria apart. In the 1990s, researchers used the entire 16S gene for bacterial identification, mainly using a method called Sanger sequencing. However, this older method was slow and could only handle a small number of samples. Then came second-generation sequencing technologies, like pyrosequencing and Illumina, which made it possible to read DNA much faster and in larger quantities, commonly used due to its cost-effectiveness and ease of implementation. Limited by resolution at species level and inability to provide functional insights. Some bacteria from the same genus (like Streptococcus or Escherichia/Shigella) have very similar 16S rRNA gene sequences, making it hard to tell them apart. This matters because some species are linked to disease, while others are linked to health. To identify bacteria more accurately, it’s important to classify them at the species level, which is easier when we analyze longer DNA reads. Here are some latest technologies that are much more efficient.

#### PacBio HiFi reads (high-fidelity)

Produces long reads (up to full-length 16S) with very high accuracy (≥99.9%). Recent studies have demonstrated that full-length 16S sequencing can reveal gut microbiome signatures predictive of metabolic dysfunction-associated steatotic liver disease in children with obesity, highlighting its potential for clinical biomarker discovery[21].

#### Oxford Nanopore (Q20 + chemistry & Kit 14)

Oxford Nanopore Technologies offers portable, real-time sequencing devices (e.g., MinION, Flongle) capable of generating long-read 16S sequences. With recent improvements in chemistry (Q20 + accuracy, Kit 14), Nanopore sequencing now achieves near-full-length 16S reads with substantially improved accuracy and throughput, making it suitable for point-of-care and resource-limited settings. A 2024 study demonstrated high accuracy and throughput for near full-length 16S amplicon sequencing, supporting its utility in rapid microbiome diagnostics[22].

#### Loop genomics synthetic long-read technology

Reconstructs full-length 16S rRNA genes from short-read Illumina platforms using unique molecular barcoding with low error rates and cost-effective workflows[23].

### Metagenomics (whole-genome sequencing)

Allows comprehensive profiling of microbial communities, including bacteria, viruses, and fungi[24]. Offers functional analysis by identifying genes related to metabolism, resistance[25], and virulence[26]. However, shotgun metagenomics is significantly more expensive and computationally demanding than 16S sequencing, requiring substantial bioinformatics expertise and infrastructure for data analysis. The complexity of assembling and annotating metagenomic data, combined with challenges in distinguishing closely related strains, can limit its accessibility for routine clinical use. Moreover, the large volume of sequence data generated necessitates standardized pipelines to ensure reproducibility and comparability across studies.

### Metabolomics and functional metagenomics

Recent advancements in multi-omics, especially integrating metabolomics and functional metagenomics, are revolutionizing our understanding of the microbiome beyond simple taxonomic surveys. By quantifying microbial metabolites like short-chain fatty acids (SCFAs), lipids, and amino acids, we gain powerful clues into how gut microbes actively shape host metabolism, inflammation, and disease states. For example, scientific reports have traced metabolite changes back to specific microbial species in both clinical (e.g., acute pancreatitis) and healthy (e.g., athlete) cohorts.

Simultaneously, functional metagenomic techniques allow researchers to isolate, clone, and express microbial genes directly from environmental DNA. This reveals not only which genes are present (e.g., antibiotic resistance, metabolic pathways) but also whether they are active and impactful shining light on gene–function relationships tied to diet, immunity, and host health .

Understanding the diversity within and between microbial communities offers powerful insights into how microbiomes influence health and disease. Alpha diversity which measures how many species are present in a single sample and how evenly they are distributed helps us gauge a community’s richness and balance. In clinical contexts, low alpha diversity often signals dysbiosis and is linked to inflammatory or metabolic conditions[27].

On the other hand, beta diversity which compares community composition across multiple samples enables us to see how microbiomes differ between health states or treatment groups. For example, measures like Bray–Curtis or UniFrac can reveal distinct microbial patterns that distinguish diseased individuals from healthy controls[28]. Proper application and interpretation of diversity metrics require careful consideration of study design, sample size, sequencing depth, and statistical power, as these factors can significantly influence results and their clinical relevance.

Table 2 summarizes the major microbial analysis techniques used in gut microbiome research, highlighting their strengths, limitations, and clinical relevance. 16S rRNA gene sequencing offers cost-effective bacterial profiling suitable for large-scale studies but has limited species-level and functional resolution. Shotgun metagenomics provides comprehensive taxonomic and functional insights, detecting bacteria, viruses, and fungi simultaneously, though it requires high cost and complex bioinformatics. Metabolomics reveals real-time metabolic activity of gut microbes and their influence on host physiology but demands advanced analytical interpretation. Diversity indices (α and β) give a broad ecological overview of microbial variation across health and disease states, serving as valuable indicators of dysbiosis. Collectively, these approaches provide complementary perspectives that enhance diagnostic precision and guide personalized, microbiome-based therapeutic strategies in GI disorders.

**Table 2:** Comprehensive comparison of microbial analysis techniques: strengths, limitations, and clinical relevance in gastrointestinal disorders.

Analysis technique	Strengths	Limitations	Clinical relevance
16S rRNA gene sequencing	Cost-effective, requires relatively low computational power, allows taxonomic identification of bacterial genera across diverse samples.	Limited resolution at species level; not suitable for functional or metabolic characterization.	Enables rapid and affordable profiling of gut microbiota composition, useful for identifying dysbiosis in conditions like IBD or IBS.
Metagenomics (whole-genome sequencing)	Provides comprehensive taxonomic and functional profiling; detects bacteria, viruses, and fungi simultaneously.	High sequencing cost and data analysis complexity requiring advanced bioinformatics tools.	Allows identification of microbial genes linked to disease phenotypes and antimicrobial resistance, supporting personalized therapeutic strategies.
Metabolomics	Reveals real-time functional activity of the microbiome and its metabolic impact on host physiology.	Complex data interpretation; requires integration with other omics data for context.	Identifies microbial metabolites such as SCFAs, bile acids, and tryptophan derivatives relevant to gut–brain and immune interactions.
Diversity indices (α and β diversity)	Simple and rapid comparative tool; provides ecological overview of microbial variation across conditions or interventions.	May not reflect true metabolic or pathogenic significance.	Useful in assessing microbial diversity shifts in diseases (e.g., reduced diversity in obesity, Crohn’s disease, or antibiotic exposure).

IBD, inflammatory bowel disease; IBS, irritable bowel syndrome; SCFA, short-chain fatty acid.

While these technologies enable increasingly detailed characterization of microbial communities, their clinical value ultimately depends on how effectively such data can be translated into reliable diagnostic tools for GI disease.

## Microbiome as a diagnostic tool in gut disorders

The IBDs, ulcerative colitis (UC) and Crohns disease, are chronic inflammatory disease of GI tract affecting increasing number of individuals. Large cohort meta-analyses indicate that combined microbial signatures in IBD provide moderate diagnostic discrimination, with area-under-the-curve (AUC) values generally ranging from approximately 0.70 to 0.80 when distinguishing IBD patients from healthy controls, although diagnostic performance remains heterogeneous across populations and study methodologies^[^11,19,29^]^.

Considering the constantly increasing prevalence in the developed countries, and the rapidly surging incidence in the developing countries, the disability-adjusted life year and burden of disease are on the increase with much global concern[11]. For CRC, colonoscopy and FIT remain the gold standards for early detection, whereas microbiome-derived markers are still considered adjunctive tools under investigation[3]. Meta-analyses indicate that certain bacterial species, including Fusobacterium nucleatum, Bacteroides fragilis, and Parvimonas micra, demonstrate moderate discriminative ability for CRC detection (AUC ≈ 0.78–0.85); however, these findings have not yet achieved the sensitivity or specificity required for clinical deployment[30]. Collectively, these studies suggest that while the gut microbiome holds diagnostic potential, its current role is complementary, enhancing existing methods rather than replacing them. Integrating microbial biomarkers with non-invasive modalities such as metabolite profiling, machine-learning algorithms, or host genomic data may ultimately yield more accurate and clinically practical diagnostic models. Despite significant advances in sequencing and computational analysis, no microbiome-based diagnostic test has yet achieved clinical validation or regulatory approval for use as a standalone diagnostic tool in IBD, IBS, or CRC[19]. When compared to current standards such as FIT and colonoscopy, microbiome-based diagnostics remain largely investigational. FIT, which detects human hemoglobin in stool, demonstrates a sensitivity of approximately 79% and specificity of 94% for CRC detection, while colonoscopy offers sensitivity above 95% for advanced neoplasia under optimal conditions[31]. In contrast, metagenomic or 16S-based microbial biomarkers – including Fusobacterium nucleatum and Bacteroides fragilis – achieve diagnostic accuracies ranging from 70% to 85% (AUC 0.78–0.85) in research settings but lack external validation and regulatory approval[30]. Consequently, microbiome profiling currently serves as a complementary approach rather than a replacement for FIT or colonoscopy, potentially enhancing non-invasive risk stratification when integrated with existing screening modalities[19].

Most microbiota studies are based on amplicon sequencing of the bacterial and archeal 16S rRNA gene. However, the Microbiome Quality Control project has shown that variation in each step of the laboratory and bioinformatics pipeline introduces different biases in the resulting microbiota datasets. Pipeline challenges include sample storage, DNA extraction method, primer choice, sequencing depth, and bioinformatics quality control procedures. A large, combined sample size increases the chance of identifying robust and consistent IBD signatures across geographic locations, enables adjustment for individual technical choices, and increases the statistical power to detect new genera associated with IBD. Therefore, the aim of the present study was to identify, combine, and re-analyze original 16S rRNA amplicon sequencing data from internationally available cohorts of patients with IBD and healthy individuals, while taking technical variation between studies into account, to provide well-powered and robust microbiota signatures for IBD[29].

The human microbiome may identify microbial biomarkers and aid in the diagnosis of various diseases such as IBD, colon cancer, and many others but none are yet FDA-approved. The manuscript integrates recent advances in microbiome research, highlights how microbial signatures can transform early cancer detection and personalized treatment strategies. For significance, the study highlights the critical need for noninvasive, accurate, and tailored diagnostic methods in oncology care. By integrating microbial biomarkers with conventional diagnostic methods such as liquid biopsies and machine learning algorithms, the article offers a forward-looking perspective on solving diagnostic and therapeutic challenges in oncology and IBD.

Beyond diagnosis and risk stratification, these disease-associated microbial patterns also provide a rationale for therapeutic strategies aimed at modulating the gut microbiome.

## Personalized therapeutics: Microbiome-guided interventions

### Microbiome-informed dietary modulation

Diet plays a crucial role in the regulation of the microbiome in the gut, and this influence determines which microorganisms are beneficial and how the body responds to disease. Life’s early stages are particularly important, as the initial sources of nutrition have a significant impact on the microbiota’s development, human milk oligosaccharides, and immune components like SIgA play vital roles in shaping microbial diversity and immune development. The changes within 24 h of a dietary shift are significant, but the microbiome can recover to its original state with a dietary rest. While microbiota restoration (reintroducing lost microbes) has been proposed, a more practical strategy involves increasing dietary fiber, minimizing food additives, and eating seasonally to support beneficial microbial communities. The modern lifestyle, especially in urban areas, has led to a significant difference from the ancestral microbiome. The mode of delivery and breastfeeding have an effect on the infant’s gut colonization; overall, diet is more important in regard to altering the microbiome than genetics or antibiotics[32]. Other researches amplify the story by relating fiber consumption to the microbiome’s shifts that affect health in the lungs, demonstrating that diet’s influence is far greater than the gut[33]. Human genetics also influence gut microbiome composition, particularly highlighting the heritable bacterium Christensenella minuta, which is linked to lower BMI. When introduced into mice, it reduced weight gain – suggesting that our genes can shape gut microbes in ways that affect metabolism and potentially disease risk[34].

**Figure 2.**
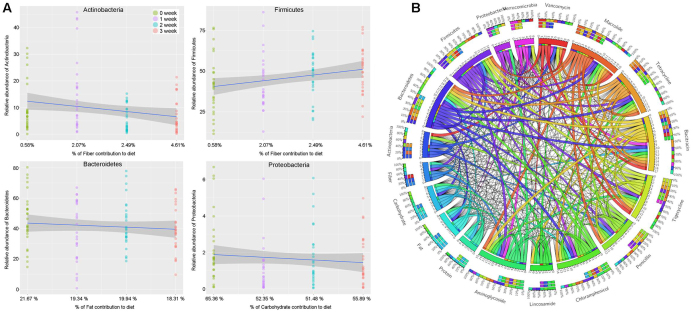
(A) Association between dietary macronutrient intake (fiber, fat, carbohydrate) and the relative abundance of major gut microbial phyla over 3 weeks. (B) Circular chord diagram showing interactions between microbial taxa, antibiotics, and dietary components, illustrating complex microbial-environmental relationships[35].

This study found that switching staple carbohydrates (wheat, rice, oats) in Mongolian adults rapidly reshaped their gut microbiota and metabolic functions. Wheat and oats boosted bifidobacteria, while rice suppressed them. Microbial groups showed distinct preferences for different carbs, and changes in bacteria mirrored shifts in gut viruses. The findings in Fig. 2A and B[35] highlight how diet can swiftly rewire gut ecosystems.

Elimination diets (e.g., low-FODMAP, gluten-free, lactose-free) may reduce symptoms but can reduce beneficial taxa such as Bifidobacterium and Faecalibacterium prausnitzii, potentially worsening dysbiosis. In contrast, the Crohn’s Disease Exclusion Diet with partial enteral nutrition has shown greater potential to improve microbial balance and support remission[36].

Different types of carbohydrates, from soluble fibers to resistant starches and oligosaccharides, each play a unique role in sculpting the gut microbiome. Using innovative statistical methods, the study shows that specific carbs can increase beneficial bacteria like Bacteroides, Actinobacteria, and Lactobacillus, while others reduce potentially harmful taxa. These findings underscore the deep connection between our diet and microbial health, suggesting that the carbs we choose may subtly influence everything from immunity to disease risk[37].

Microbiome-informed dietary personalization is no longer theoretical. However, follow-up analyses indicate that the predictive accuracy and clinical benefits of AI-guided dietary interventions often attenuate over time, with reductions in performance and symptom improvement observed beyond approximately 6–12 months in the absence of sustained adherence and ongoing dietary reinforcement^[^38,39^]^. From AI-tailored meals that transform IBS symptoms severity reductions of ~ 39% in IBS and remission rates of ~ 72% in diabetes context**^[^**38,39^]^, to n-of-1 trials[40] generating macronutrient plans, individualized microbiome analysis is sharply enhancing diet efficacy and patient outcomes. However, despite their promise, these strategies are not without challenges. These AI-guided dietary platforms face several limitations. Their predictive accuracy depends on the quality and diversity of training datasets, which may not reflect global dietary habits[39]. Most models lack large-scale clinical validation and are constrained by the dynamic, time-varying nature of the human microbiome. Moreover, many deep-learning systems limits interpretability and clinician trust, while continuous data collection raises privacy and security concerns[41]. Addressing these limitations will be essential to ensure reproducibility, transparency, and safe clinical translation of AI-driven nutrition technologies. Long-term adherence often wanes due to dietary restrictions, lack of support, or nutritional inadequacies. Moreover, the safety of prolonged restrictive diets especially when unsupervised raises concerns about potential micronutrient deficiencies and impaired gut microbial diversity. Psychological and behavioral interventions also show potential, yet adherence remains a significant barrier, particularly when interventions are self-guided or technology-mediated. These findings underscore a critical insight while symptom control and inflammatory reduction are increasingly achievable through personalized non-pharmacologic therapies, the success of these interventions hinges on sustained engagement, professional guidance, and individualized risk-benefit assessments.

### Precision probiotic therapy

Precision probiotic therapy involves selecting strains based on host microbiome features and disease context to restore specific functional deficits[42]. Mechanistically, precision probiotics exert effects through short-chain-fatty-acid production, competitive exclusion of pathogens, reinforcement of mucosal tight junctions, and modulation of T-cell differentiation. Recent genomic and metabolomic mapping allows identification of probiotic strains with specific anti-inflammatory, neuroactive, or metabolic profiles suitable for distinct clinical conditions. Unlike traditional over-the-counter probiotics that offer generalized blends with inconsistent outcomes, precision probiotics are carefully selected based on a patient’s unique microbiome profile, aiming to restore specific imbalances in microbial composition and function. Studies now show that this approach can lead to meaningful clinical benefits: for example, Yang *et al*[43] demonstrated that symptom-specific probiotic blends like ConstiBiome and SensiBiome significantly improved bowel-related symptoms in patients when matched to their stool patterns. and recent systematic reviews confirm that probiotics provide modest yet statistically significant symptom improvement in IBS (57 % *vs* 35 % for placebo) and a 15–20 % reduction in relapse risk for UC[44], while other research has explored nano-encapsulation technologies to enhance the delivery and survivability of these live organisms such as nanoparticles, liposomes and hydrogels being increasingly utilized to form protective coatings around probiotics, acting as shields that protect the microorganisms from gastric acid, bile salts, and digestive enzymes. This encapsulation ensures that probiotics remain viable as they pass through the stomach and enter the small intestine, where they can begin to exert their beneficial effects[45]. A meta-analysis also further demonstrated that probiotic interventions reduced global IBS symptom risk with a relative risk of approximately 0.77 (95% CI: 0.62–0.94), indicating modest but clinically meaningful benefit compared with placebo[46].

With the rapid advancement of sequencing technologies and computational tools, clinicians now have the potential to move beyond empirical prescribing toward data-informed decision-making. By analyzing stool samples through 16S rRNA gene sequencing or shotgun metagenomics, physicians can identify specific dysbiotic patterns – such as low abundance of SCFA producers or overgrowth of pro-inflammatory taxa – and use this insight to recommend targeted probiotic strains that restore microbial balance. For physicians, this transition involves selecting suitable candidates with persistent or refractory symptoms, performing stool-based microbiome assessments, interpreting microbial patterns through validated platforms, and matching them with strain-specific probiotics shown to restore microbial balance. While challenges remain regarding accessibility, standardization, and clinician training, the integration of microbiome-informed therapeutics into clinical workflows marks a promising step toward precision medicine in gastroenterology. Moreover, most available probiotic products remain unregulated as dietary supplements rather than licensed biologicals, leading to inconsistent quality control. Precision probiotic development therefore requires standardized manufacturing, validated biomarkers of response, and well-designed RCTs before broad clinical implementation.

### FMT

**Figure 3.**
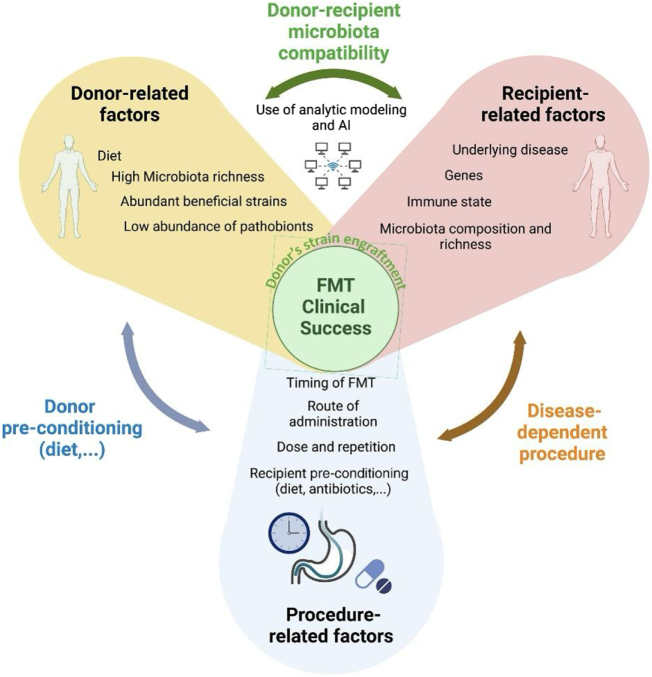
Factors that influence FMT success[34].

FMT, the process of transferring stool from a healthy donor to a patient has emerged as a powerful therapeutic strategy to restore gut microbial balance, with particularly high success rates in recurrent Clostridioides difficile infection (rCDI), where cure rates exceed 85–90%[47]. Figure 3^[^47^]^ illustrates the key factors influencing the clinical success of FMT, including donor characteristics (e.g., high microbial richness, low pathobionts), recipient conditions (e.g., disease state, immune status), and procedural variables (e.g., timing, dosage, route). Optimal donor–recipient microbiota compatibility, aided by modeling and AI, is essential for effective microbial engraftment and therapeutic outcomes.

More recently, the integration of microbiome profiling into donor–recipient matching has improved outcomes in complex conditions like UC and Crohn’s disease (CD), revealing that “super-donors” with high microbial richness and metabolic flexibility may play a key role in clinical response[48]. The term “super-donor” refers to an FMT donor whose stool produces unusually strong and consistent therapeutic benefits across multiple recipients. These donors often have a gut microbiome that is exceptionally rich in bacterial diversity and balanced in composition, with higher levels of anti-inflammatory species such as Faecalibacterium prausnitzii, Akkermansia muciniphila, and Bacteroides uniformis. Their microbiota are thought to restore gut homeostasis more effectively by increasing SCFA production, enhancing mucosal healing, and modulating immune responses. In UC, FMT helps about 25–45 % of patients reach remission, especially when stool from several donors is used or when treatment is repeated[49]. Results in CD and IBS are mixed, showing temporary benefit in some people but no long-term cure. We explore how multi-omics integration (metagenomics, metabolomics, and proteomics), AI-driven donor–recipient matching algorithms, and targeted microbial consortia may refine FMT into a fully personalized, scalable treatment. Additionally, we highlight under-investigated areas such as the role of host immune profiling in predicting FMT response, and the potential for synthetic, next-generation FMT capsules to replace invasive procedures. A recent meta-analysis found that FMT using single or pooled donors shows comparable safety profiles in UC patients, with no significant difference in adverse events. However, pooled donor FMT demonstrated a slightly better benefit–risk ratio, supporting its continued development as a standardized therapeutic approach[50]. However, FMT still has safety and legal challenges. There is a small risk of spreading infections, donor screening is not the same everywhere, and the long-term effects are still unknown[51]. Further large-scale, controlled trials are warranted to confirm these trends and explore personalization based on donor–recipient microbial compatibility. This review aims to bridge current clinical evidence with emerging technological and systems biology approaches, offering a unique lens for clinicians and researchers to reimagine the future of microbiome-based therapeutics.

## Translational challenges and clinical integration

Although microbiome research has advanced greatly, applying findings in clinics faces major challenges. Multi-omics, computational biology, and lab studies have shown disease links, but translating these into treatments or diagnostics is difficult[52]

### Individual differences and no “Healthy” standard

People have very different microbiomes due to genetics, diet, age, medications, and lifestyle[34]. Microbiomes also change over time. This makes it hard to define a “healthy” microbiome or set standard diagnostic thresholds.

**Solution**: Use long-term studies across diverse populations and machine learning to create personalized reference ranges.

### Lack of standardization

Methods vary in sample collection, DNA extraction, sequencing, and data analysis, causing inconsistent results^[^29,45^]^.

**Solution**: Standard operating procedures (SOPs), validated kits, consistent bioinformatics pipelines, and metadata reporting[18].

### Association vs causation

Most studies show correlations, not cause-and-effect. Dysbiosis may result from disease rather than cause it.

**Solution**: Mechanistic studies using multi-omics, animal models, and human trials to prove causality.

### Regulatory and safety issues

Regulations are unclear for probiotics, FMT, or microbiome products. Risks include infections, antibiotic resistance, and variable quality[53].

**Solution**: Clear guidelines, quality control, pre-market safety checks, and post-market monitoring.

### Maintaining microbiome changes

Microbiomes often return to baseline, making lasting changes hard. Diets, probiotics, or FMT may only have short-term effects.

**Solution**: Use synbiotics, personalized approaches, engineered strains, and maintenance protocols; track long-term outcomes.

### Limited long-term data

Most studies are short-term, limiting understanding of disease progression or treatment response.

**Solution**: Large, long-term cohort studies with frequent sampling and multi-omics integration.

### Multi-omics and precision medicine

Single data types give limited insight. Integrating genomes, transcriptomes, proteomes, and metabolites helps identify functional targets.

**Solution**: Collaborative multi-omics studies with AI and network analysis to guide precision medicine.

### Clinical implementation challenges

Barriers include high costs, lack of decision support tools, limited clinician training, and low insurance coverage.

**Solution**: Develop point-of-care tests, decision tools, training programs, and health-economic evidence to support adoption.

Despite encouraging results across dietary, probiotic, and fecal microbiota-based interventions, translating these approaches into routine clinical practice remains complex and inconsistent.

## Future perspectives and research imperatives

As microbiome research builds momentum, we urgently need to transition from small, isolated studies to large, multicenter trials that reflect real-world patient populations. This is essential to establish the clinical trust and statistical power required to evaluate microbiome-based diagnostics and therapies for conditions such as IBD, IBS, a functional disorder, and CRC, even though current microbiome biomarkers are mostly associative and not yet validated for routine clinical use. For example, a meta-analysis in IBS showed probiotics reduced global symptom risk (RR ≈ 0.77; 95% CI 0.62–0.94)[46].

Standardizing sample-collection methods (stool handling), sequencing platforms and bioinformatics is equally critical because methodological differences currently prevent reliable comparison and replication across studies. Integrating host factors – including genomics, epigenetics, immune profiles, and metabolomics – can provide a systems-level view of disease biology, helping explain why patients respond differently to treatments. Combining multi-omic data with artificial-intelligence/machine-learning approaches shows strong promise for stratifying patients and predicting responses, but we still need models that are transparent, clinically interpretable and validated at the bedside. Future research must explicitly address the modest benefits and variable effects of current microbiome therapies for example, multi-strain probiotics show benefit in UC but not yet in CD, along with safety concerns around live-microbial therapies and the lack of regulatory guidelines for microbiome-based products. Current evidence suggests that microbiome-based interventions demonstrate higher efficacy in UC, with remission rates often exceeding placebo by approximately 20–30%, whereas comparable and consistent benefit has not been reliably demonstrated in CD^[^19,44,49^]^. By systematically combining these elements, the field can move toward non-invasive, personalized tools that not only improve diagnostic precision and optimize treatment selection, but also reduce adverse effects and healthcare costs. This vision rests on carefully designed longitudinal studies, robust methodological standards and a critical appraisal of both efficacy and safety taking microbiome science from promise to practical clinical reality. Addressing these barriers is essential for moving microbiome research beyond proof-of-concept toward clinically validated, scalable solutions.

## Conclusion: Microbiome medicine on the horizon

Every flare-up, bloated episode, or unexplained symptom reflects a complex interaction between the gut, the immune system, and its vast microbial community. Modern tools such as 16S rRNA sequencing, metagenomics, and metabolomics now allow early detection of dysbiosis, prediction of treatment outcomes, and identification of therapeutic targets. Personalized approaches ranging from microbiome-informed dietary changes and precision probiotics to donor-matched FMT are rapidly moving from research into clinical reality. A structured care pathway begins by identifying patients with IBS, IBD, or CRC who do not respond to standard treatments, followed by targeted microbiome assessment to guide individualized interventions. Ongoing monitoring through symptoms, fecal biomarkers, and metabolite profiles enables adaptive treatment, while digital health tools can support patient follow-up and remote evaluation. Even in low-resource settings, diet history, symptom patterns, and treatment response can guide empirical microbiome-based care. Despite encouraging results, clinical adoption remains limited by cost, accessibility, and lack of standardization. Translating research into practice will require validated diagnostic platforms, regulatory clarity, and infrastructure for integrating microbiome testing into healthcare systems. This review focuses not just on what the science demonstrates, but also on how we might apply it, how clinicians, researchers, and care teams can incorporate microflora-guided medicines into everyday practice. Looking ahead, combining microbial ecosystem findings with genetics, immunological profiling, and perhaps artificial intelligence could aid in the development of a fully individualized model of care. Clinical translation will require validated diagnostic platforms, standardized methods, regulatory clarity, and infrastructure to integrate microbiome testing into routine care. Future progress will likely depend on combining microbiome profiling with host genomics, immune phenotyping, and interpretable machine-learning models.

## Data Availability

No data are available.

## Research registration unique identifying number (UIN)

- https://www.clinicaltrials.gov/ – for all human studies – free.
- http://www.chictr.org.cn/index.aspx – for all human studies –free.
- https://www.researchregistry.com/ – for all human studies –charge.
- https://www.isrctn.com/ – for all human studies – charge.
- https://www.crd.york.ac.uk/prospero/ – for systematic reviews – free.
- There are many national registries approved by the UN that can be found here.

Once registered, you will need to submit your assigned Unique Identifying Number (UIN) from your registration body as a mandatory part of your submission.
